# Supplementary material for: Three-dimensional localization and tracking of chromosomal loci throughout the Escherichia coli cell cycle
Source: Commun Biol. 2024 Nov 5;7:1443. doi: 10.1038/s42003-024-07155-9 (PMC11538341; doi:10.1038/s42003-024-07155-9)
Supplement: Supplementary file 2 — Supplementary Information [file 42003_2024_7155_MOESM2_ESM.pdf]

Supplementary Information

A.1 Emitter sampling using cell segmentation and background distributions

Phase-contrast images (1041 x 1302 pixels) are used to obtain segmentation masks using the Omnipose method previously described in [1]. Random crops of 128 x 128 pixels from the phase-contrast images (Supplementary Fig. 1a) are segmented to generate segmentation masks (Supplementary Fig. 1b), which are in turn used to simulate the training data. We set an average number of ~2 emitters per cell and randomly sample the emitters' center pixel locations (Supplementary Fig. 1c) using a Bernoulli trial with a fixed probability (p) of finding an emitter in that pixel. This probability is calculated using the segmentation mask (Fig. 1b) and average area of cells.

XY offsets of the emitters from the center of the pixels chosen to have emitters are uniformly sampled between [-0.5, 0.5] (Supplementary Fig. 1d-e), while z is sampled uniformly between [-700, 300] nanometers (Supplementary Fig. 1f). PSF images sampled using a spline model (SI section A.3) are 40 x 40 pixels in size and are normalized to 1 before multiplying with the photon counts. The simulated PSF (Supplementary Fig. 3a) at 0 nm has $\sim$ 2.1 % of the photons distributed over the stack at the brightest pixel, while at -500 nm the PSF has $\sim$ 0.5 % of the photons at the brightest pixel. We set the photon counts of the emitters to be in between 750 and 3000 to cover for this variation in photon distribution over the PSF pattern. Photon counts are uniformly sampled from this range to match the mean of the gray level of experimental data. Each emitter image is then placed on a larger image (Supplementary Fig. 1g), 128 x 128 pixels in size. We model the cell background (Supplementary Fig. 1h) as a function of distance from its boundary (SI section A.2). Background photons are sampled from gamma distributions parameterized based on the distance from the cell boundary (SI sections A.2). Emitters' PSF image is added to sampled background photons images and passed through the camera noise model to generate the final simulated image (Supplementary Fig. 1i). Camera noise is sampled from the camera model where the noise distribution parameters are calculated per pixel during camera calibration (SI section A.4). As we only use a smaller region (128 x 128 pixels) of the larger area of the images (1041 x 1302 pixels), the locations of the smaller regions are encoded using the CoordConv strategy [2,3] (Supplementary Fig. 1j-k) and are used as inputs to the network.

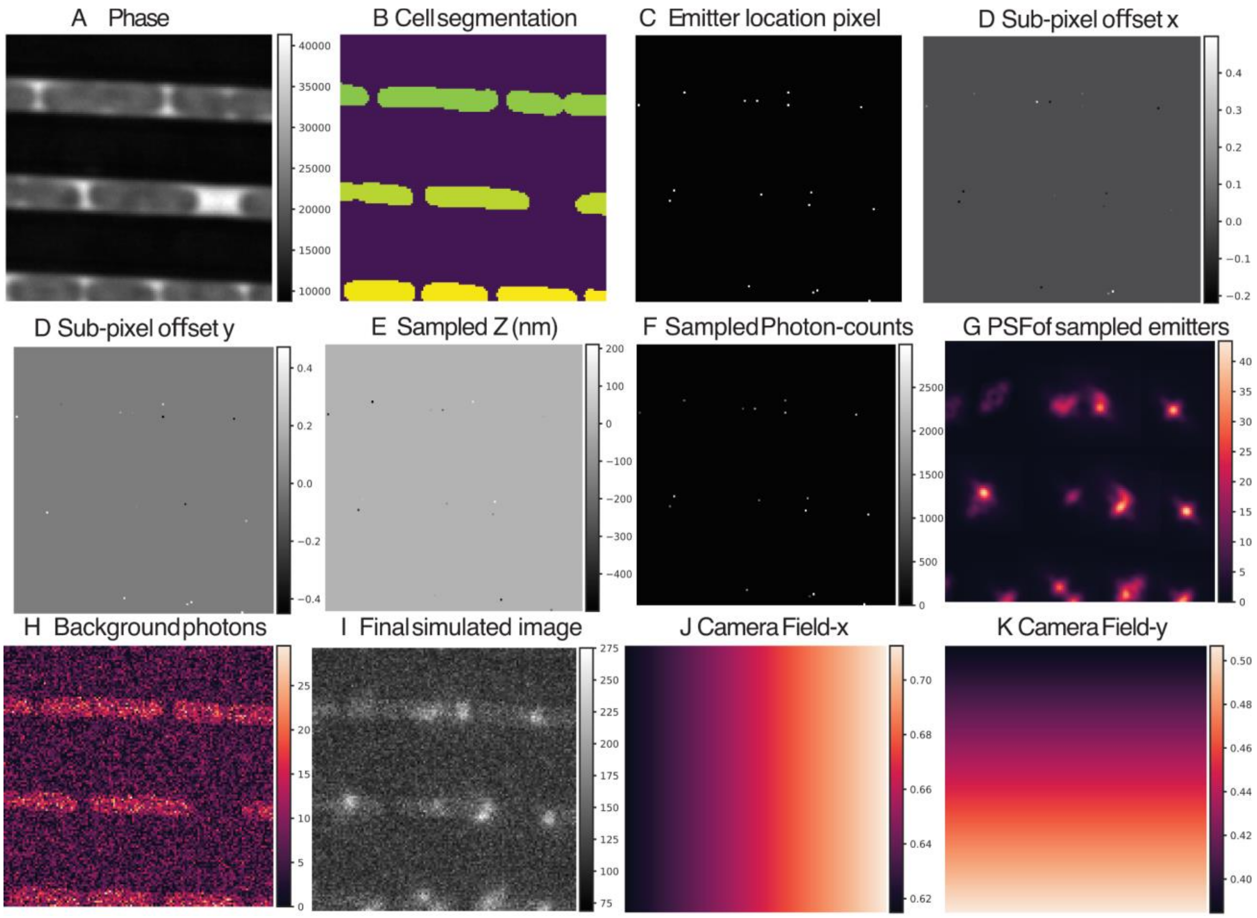

Supplementary Fig. 1: Emitter sampling and simulation of image generation of a 128x128 image. a, Phase-contrast image **b,** Cell segmentation mask **c,** Sampled emitter pixels' centers **d-g,** Sampled x, y, sub-pixel offsets from the center of the pixel. z-position sampled in range [-700, 300] nm, photon counts in the range [750-3000] **g,** Photons of all emitters overlayed onto a single image **h,** Sampled background photons from gamma distributions of background photons **i,** Final images with emitter and background photons added and converted to gray level after adding pixel-dependant camera noise **j-k,** XY field of the sub-region on the camera ROI with origin at [800, 400]. Camera ROI is of size 1041 x 1302 pixels.

A.2 Cell background estimation

Phase-contrast images are segmented to obtain cell segmentation masks after transforming phase-contrast images to the same size as fluorescence images. Cell segmentation labels are dilated by 1 pixel. Pixels on each image are masked using cell segmentation into two categories, inside and outside the cells. Pixels outside cells are used to estimate gamma distributions of background photons coming from the chip (Supplementary Fig. 3a). Pixels inside the cells are used to estimate background distributions as a function of distance to the boundary of a cell. Inside cells, only pixels less than the 75th percentile are used to estimate background, removing most potential emitters (Supplementary Fig. 2). Collections of pixels from each image at varying distances from the boundary are used to fit gamma distributions, whose mean and variance are used to sample realistic cell backgrounds during simulations. Supplementary Fig. 3b shows fits of data and distributions. This procedure is repeated over 300 images and variation of means of the fitted distributions is shown in Supplementary Fig. 3c. From this, we

conclude that background can be approximated as a function of distance from the boundary. Mean and variance masks are created for each cell mask used to sample emitters using the average values over all 300 images. Supplementary Fig. 3d shows these maps for an example cell mask. Sampled background gray level is converted to photons and is used for simulations (Supplementary Fig. 1h).

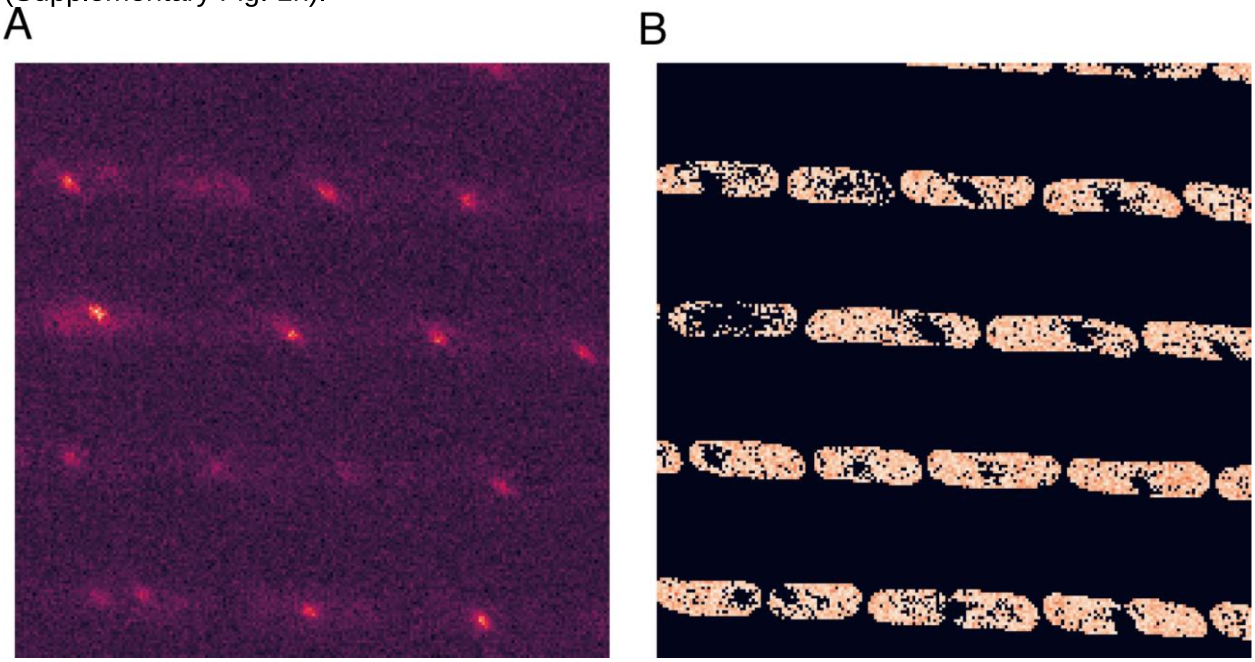

Supplementary Fig. 2: a, Fluorescent image with emitters. **b,** Image with emitters removed to estimate cell background.

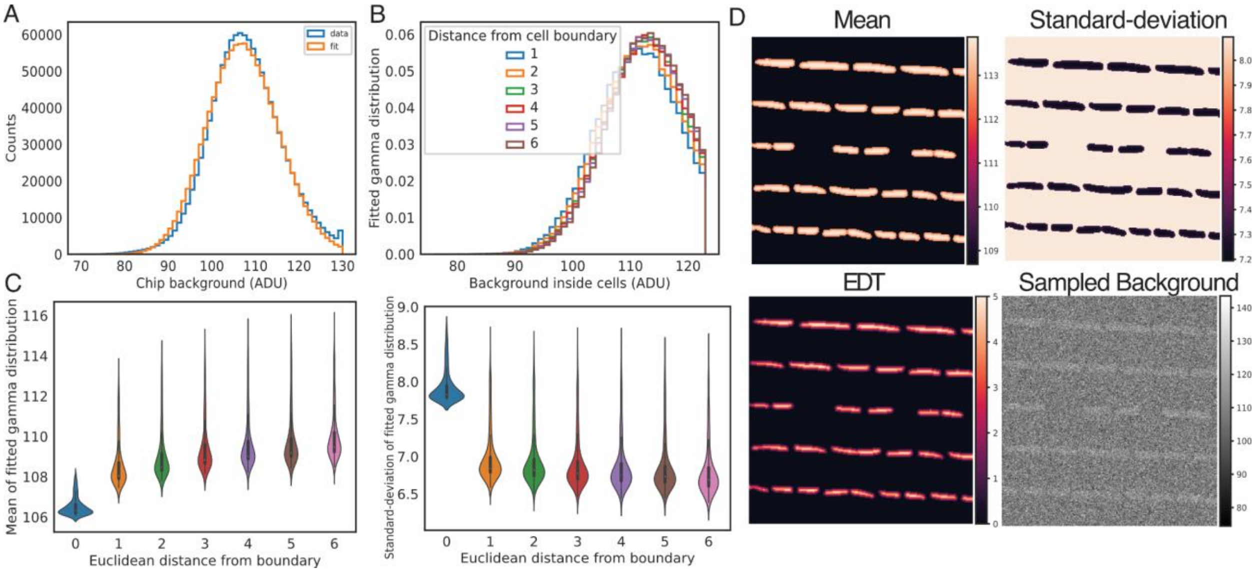

Supplementary Fig. 3: Cell and fluidic chip background for chromosome dots a, Chip background distribution and fit over one image. **b,** Distribution fits as a function of Euclidean-distance from the boundary for pixels inside cells **c,** Mean and standard deviation of the fitted gamma distribution over 300 images as a function of EDT. EDT=0 corresponds to chip background. **d,** Mean and variance over an example cell mask and corresponding sampled

background using distributions from Supplementary Fig. 3c.

A.3 Experimental astigmatic point spread function sampling

Emitter images are sampled using DECODE’s spline sampling function [4]. Supplementary Fig. 4a shows the PSF shape as a function of Z. Supplementary Fig. 4b shows the Cramer-Rao lower bound (CRLB) of the localization precision achievable from the cubic spline model of the PSF in the low noise (high signal-to-background) regime with a uniform background model (1875 photons for the emitters and 20 photons for the background). To obtain more uniform localization precision on most emitters in the experimental data, z-range of imaging was confined -700 nm to 100 nm.

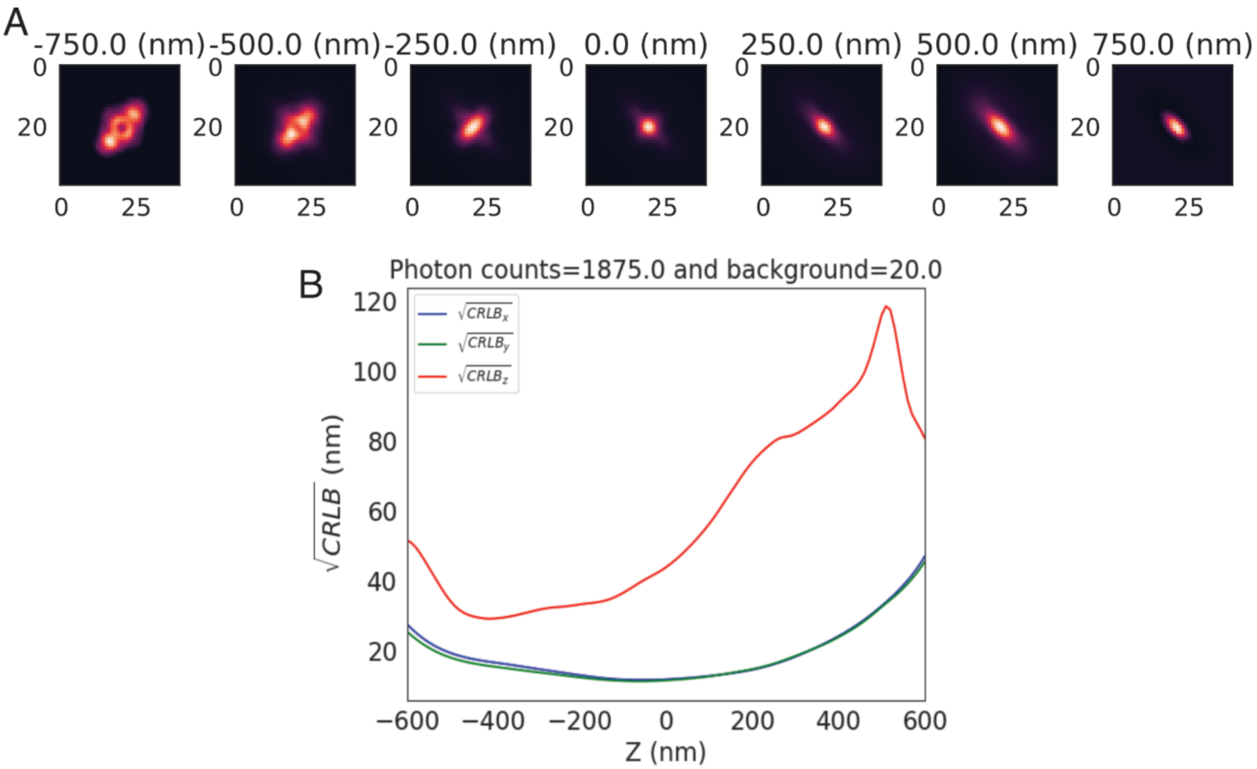

Supplementary Fig. 4: Astigmatic point spread function sampling. a, Normalized PSF stack in the YFP imaging channel obtained from averaging 50 fluorescent beads. Each image of the PSF is sampled from the CSpline model where emitters have varying z from -750 nm to +750 nm placed at the center of the image and normalized such that each image sums to 1.0. **b,** CRLB of the localization precision in x, y and z using the spline model at signal-to-background ratio similar to chromosomal loci emitters. This represents the best achievable localization precision possible with this PSF and unbiased model.

A.4 sCMOS camera model

The camera noise model is approximated using experimentally determined parameters and is used to convert photons to image units (gray level) and vice-versa. Photon statistics, pure light intensity fluctuations, is modeled as a Poisson process. Conversion from photons to electrons by absorption of photons in the sCMOS sensor is accounted for by an absorption probability that scales with the mean of the Poisson process.

Different sources of noise considered are shot noise, read-noise (RN) and thermal noise (TN). Shot noise accounts for Poissonian distribution of photons hitting the camera pixels. Read-noise accounts for noise in reading electrons, and thermal noise accounts for electrons generated due to thermal excitation.

If $\lambda_{ph,k}$ is the mean number of photons collected in pixel k, and q_e is quantum efficiency at a given imaging wavelength, then the mean number of electrons from pixel k, is

$$\lambda_{e,k} = \lambda_{ph,k} q_e.$$

The number of electrons s_k accumulated at pixel k during any camera exposure follows a Poissonian distribution with mean given by $\lambda_{e,k}$:

$$p(s_k) = \frac{\lambda_{e,k}^{s_k} e^{-\lambda_{e,k}}}{s_k!}.$$

The pixel-wise RN and TN for sCMOS cameras are typically modeled as Gaussian distributions with variances σ_{RN}^2 and $\sigma_{TN}^2 t$, where t is exposure time in seconds. In our modeling we estimate them using dark-noise images (SI A.5) and combine them into one Gaussian distribution with variance $\sigma_{N,k}^2 = \sigma_{RN,k}^2 + \sigma_{TN,k}^2 t$ for each pixel k. The reading and thermal noise N_k for each pixel k is thus sampled from the Gaussian distribution as

$$p_{e,k}(N_k) = \frac{1}{\sqrt{2\pi\sigma_{N,k}^2}} exp\left(-\frac{N_k^2}{2\sigma_{N,k}^2}\right).$$

and is added to s_k. Gain (G, gray level/e-) is used to convert electron signal to image gray values and a predefined offset value of 100 is added to the counts to obtain the final image values (I_k):

$$I_k = G\,(s_k + N_k) + offset.$$

A.5 Camera calibration and camera noise sampling

5000 dark images at varying exposure times of 25 ms, 50 ms, 75 ms, 150 ms, 300 ms over the ROI (1041 x 1302 pixels) on the camera (3200 x 3200 pixels) are used to calibrate camera offset, gain, pixel-wise read noise and thermal noise parameters using the Accent plugin [5] in Fiji. Supplementary Fig. 5a shows the distribution of gain values estimated by the plugin over the camera ROI. Due to the noisy nature of gain-estimation using this method, we use the median value of gain (~ 2.58) instead of using pixel-wise gain values in the camera model. The plugin also outputs pixel-wise standard deviation ($\sigma_{N,k}$) at 150 ms in gray level units. Supplementary Fig. 5b shows the distributions combined read-noise and thermal noise in the standard e- units used by CMOS camera manufacturers. We use the median gain value wherever there is a conversion from gray level to e- units required. Supplementary Fig. 5c shows a camera noise over a smaller ROI on the camera.

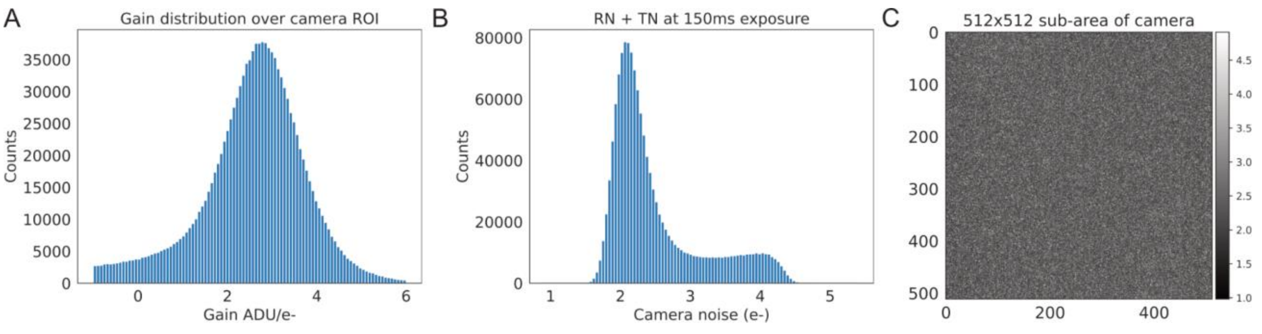

Supplementary Fig. 5: Camera gain, camera noise histograms and maps of camera noise. **a,** Gain distribution over the full camera ROI (1041 x 1302 pixels) **b,** Histogram of read noise+thermal noise standard deviation distribution at 150 ms exposure over all the pixels in the camera ROI (1041 x 1302 pixels). **c,** Read noise + thermal noise over a smaller sub-region on the full ROI in electrons to show absence of structured patterns.

A.6 Network training performances and evaluations of trained models

For an input image of size 128 x 128 pixels containing emitters and corresponding camera XY-field, the localization network predicted probabilities, XY sub-pixel offsets, z and photon counts along with their σ's and PSF of all predicted emitters in the image. These values are scaled to nanometers for x, y, z and real values for photon counts. The pixel-wise probabilities produced by the network are processed to localize the emitters after summing probabilities in the neighboring 4 pixels. The summed probabilities are thresholded (p>0.8) to find the local maxima. Each emitter is matched to ground truth emitters in a radius of 250 nm around the predicted localization. A total of ~21000 emitters simulated in 30 evaluation images of full camera FOV, with a 1041 x 1302 pixel size were used to calculate these metrics during training of the network. A final precision ~1.0 and recall of ~0.8-0.95 was achieved after 20000-40000 iterations of training with a batch size of 64 per iteration for both chromosomal loci emitters. Supplementary Fig. 6 shows the convergence of the loss function, RMSE-x, RMSE-y, RMSE-z, precision (TP / TP + FP) and recall values (TP / TP + FN) for one model trained for localization of chromosomal loci. Evaluation of any biases in learning requires examining distributions of residuals of the simulated ground-truth emitters and model predictions. Supplementary Fig. 7a-d shows four distributions for deviations of x, y, z and photon count predictions from the matched ground-truth emitters. Sub-pixel offset prediction distributions are shown in Supplementary Fig. 7e-f. Means of deviations of localizations predicted by the network are close to 0, indicating that the predictions are not biased in the overall z-range over which the network is trained on.

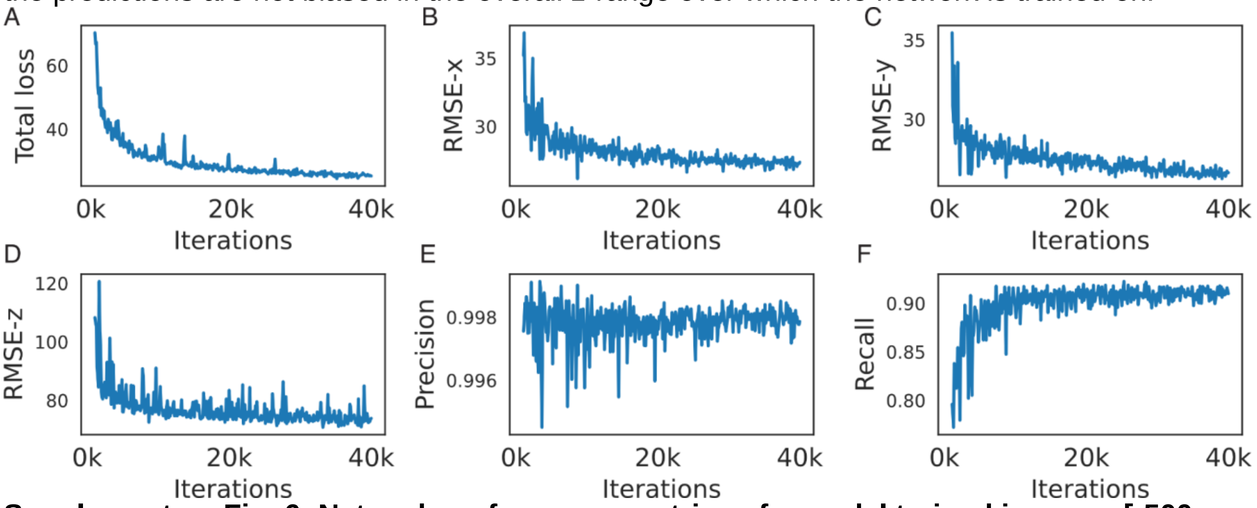

Supplementary Fig. 6: Network performance metrics of a model trained in range [-500, 500] nm of the PSF as function of training-loop iteration number. a, Overall loss value **b,** RMSE x **c,** RMSE-y **d,** RMSE-z (E) Precision (TP / TP + FP) (F) Recall (TP / TP + FN).

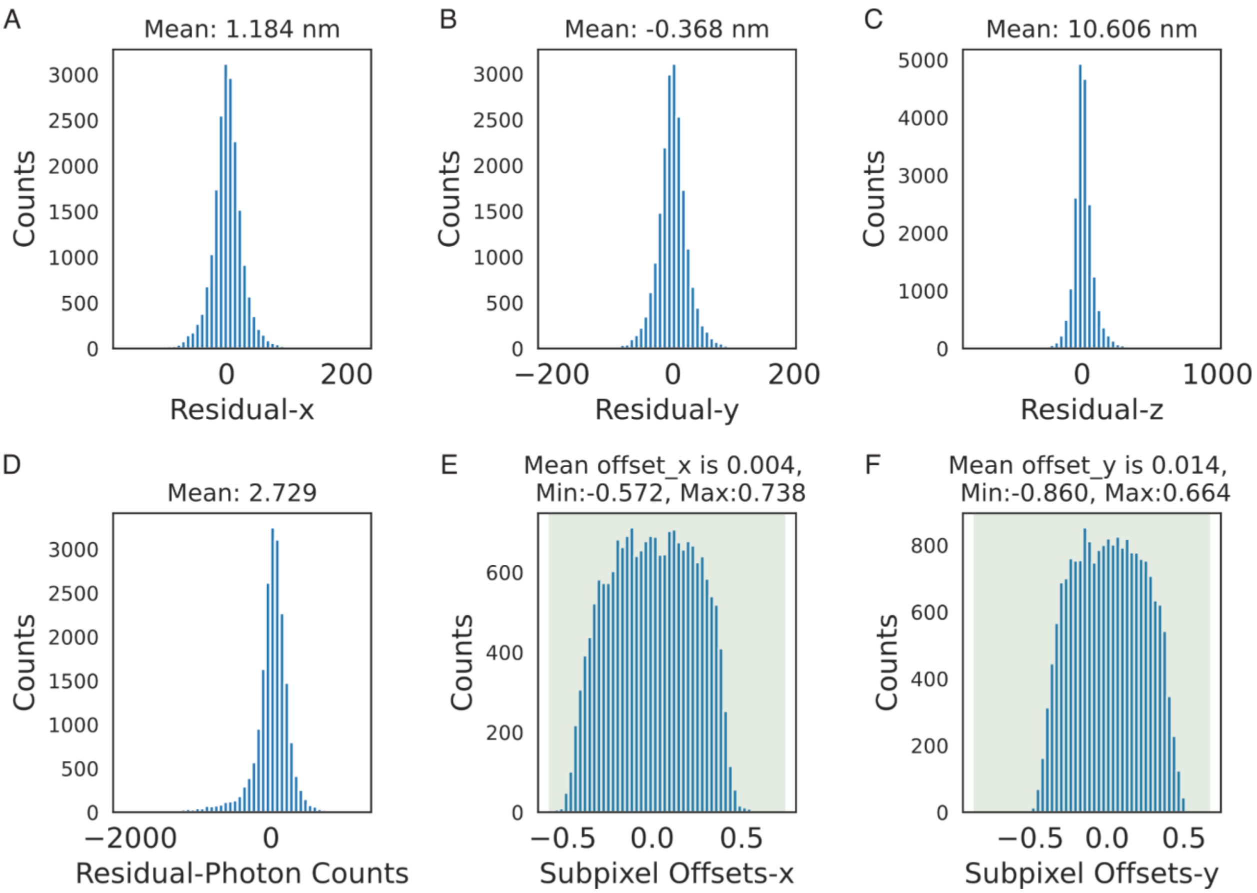

Supplementary Fig. 7: Distributions of deviations between matched ground-truth emitter locations and localizations predicted by the model. **a,** x-deviation **b,** y-deviation **c,** z-deviation **d,** photon count deviation **e,** Sub-pixel offset-x distribution **f,** Sub-pixel offset-y distribution.

A.7 Signal-to-background ratio calculation

Signal-to-background ratio of emitters is calculated from raw images and cell background images predicted by the network after removing the camera offset. Average cell background $I_{background}$ is calculated using the background prediction and cell segmentation mask using only pixels inside cells. Signal-to-background ratio of an emitter is calculated as $\frac{I_{signal}-I_{background}}{I_{background}}$, where I_{signal} is the raw image with camera offset removed. Maximum signal-to-background ratio over a 7 x 7 pixels ROI around the localized emitter is calculated for ~178000 emitters for the chromosomal midway loci. A similar procedure is repeated on the evaluation data used during training of the localization network. Their distributions are shown in Supplementary Fig. 8.

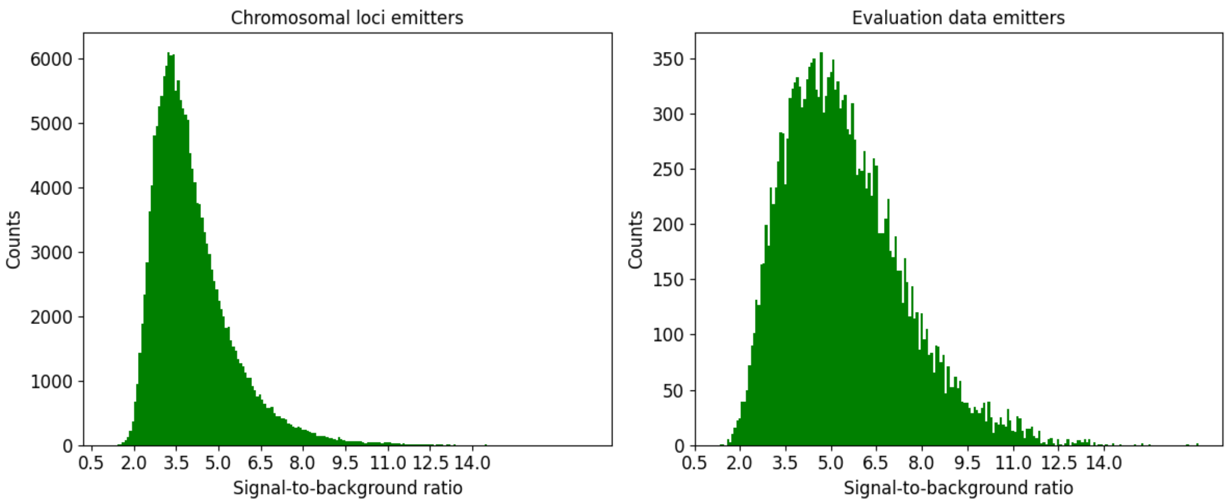

Supplementary Fig 8: Evaluating signal-to-background ratios of the emitters. Histograms of signal-to-background ratios of n=178262 emitters of chromosomal loci (left) and evaluation data (right) n=19650 emitters.

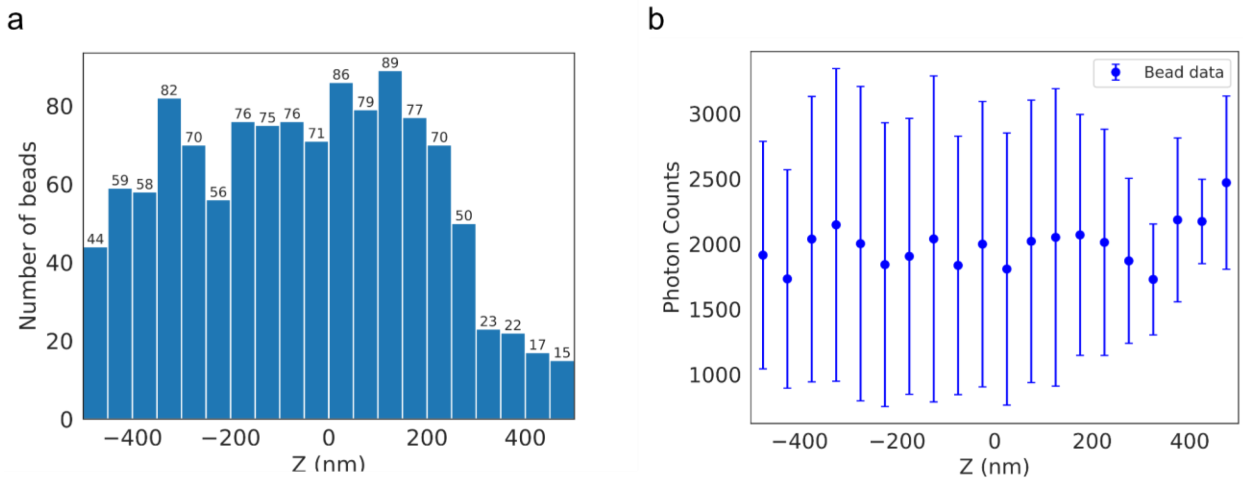

Supplementary Fig. 9: Fluorescent bead localization over the z-range. a, Number of the detected beads in each z bin. **b,** Photon counts of the detected beads in each z bin.

A.8 Locus localization uncertainties predicted by the network

The loci localizations visualized in Fig. 3 have associated uncertainties (σ_x, σ_y and σ_z) predicted by the network. These uncertainties are plotted against the internal coordinate assigned to each localization in x, y and z (Supplementary Fig. 10). The internal coordinate in x is scaled in the range [0, 1], while y is in [-1, 1] to remove the effects of variation in cell size. We expect a symmetric distribution around 0.5 for internal-x and 0 for internal-y. For the z distribution however, we expect it to be symmetric around 0 nm, if the localization precision is uniform across the operating range in height.

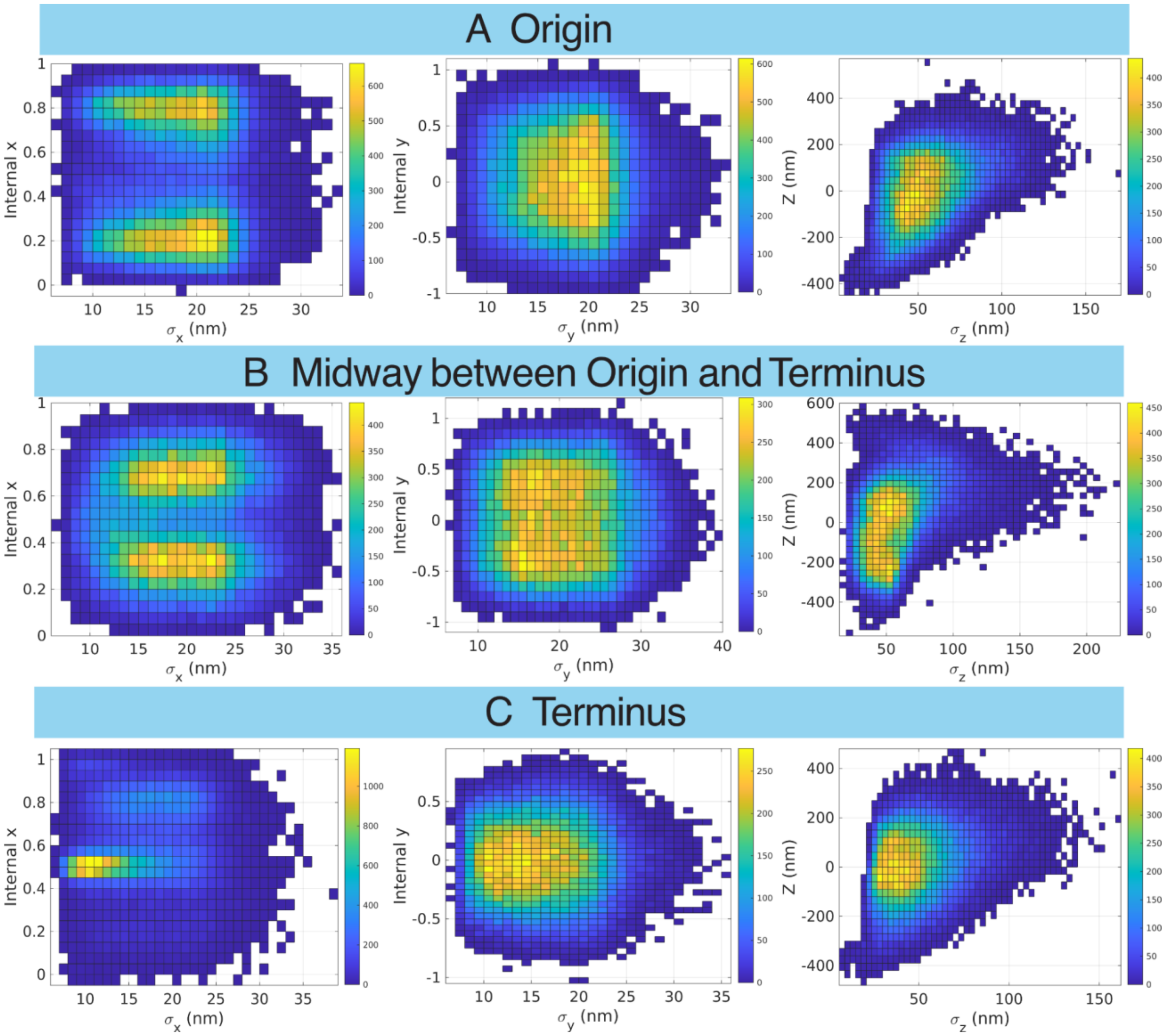

Supplementary Fig. 10: Uncertainties of emitter localizations as functions of cells' internal coordinates for x and y and real z coordinate for different chromosomal loci. The internal x coordinate is scaled to the range [0, 1], while the internal y is in the range [−1, 1]. **a,** Origin **b,** Midway between origin and terminus **c,** Terminus.

A.9 3D localizations of membrane proteins.

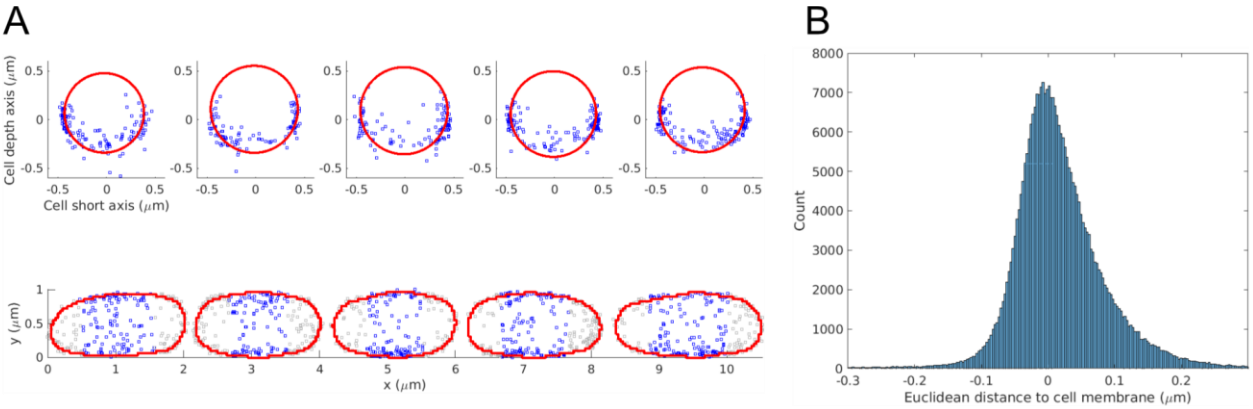

Supplementary Fig. 11: 3D localization of membrane proteins. a, Examples of 3D localization of LacY-PAmCherry in single cells. Top: Circle fit (red) fitted to y and z emitter coordinates (blue) along the cell's short axis and depth axes using the Pratt method [6] implemented in MATLAB by [7]. Bottom: Cell outlines (red) based on segmentation masks with overlaid dots, where the gray dots in the poles were discarded when fitting the circle in the top panel, while the blue dots were included. **b,** Histogram of distances to the circle fit (Supplementary Fig. 11a, top) from 2001 cells. The average standard deviation per cell is 69 nm.

A.10 3D locus localization and tracking.

To determine if loci explore the full width of the population-based location distribution, we investigated locus MSD based on 3D tracking and the variances of these distributions. In our analysis we compared the radial MSD plateau value (Fig. 4b) with the variance of the location distribution for the midway locus in the yz-plane (Fig. 4d). We also compared the long axis MSD (Fig. 4a) with the variances of the location distribution along the cell long axis (Fig. 4c). These comparisons are based on the definition of $MSD(\tau) = <(x(t+\tau) - x(t))^2>$, which can be interpreted as the variance of a zero mean distribution. The location distribution of Fig. 3b shows the localization of the locus over the cell population. Its variance includes the absolute starting position, where the locus is detected within the cell, and the spread due to the locus motion over time. The MSD, on the other hand, describes the relative motion of a single locus, as the trajectories it is based on are only related to themselves. If the MSD as a function of time lag reaches a plateau, the comparison with the population-based distribution variance can be made directly (e.g. Fig. 4d). However, if no plateau is reached on the measured time scale the generation time of the cells has to be taken into account to conclude if the locus movement will span the population-based distribution over one generation.

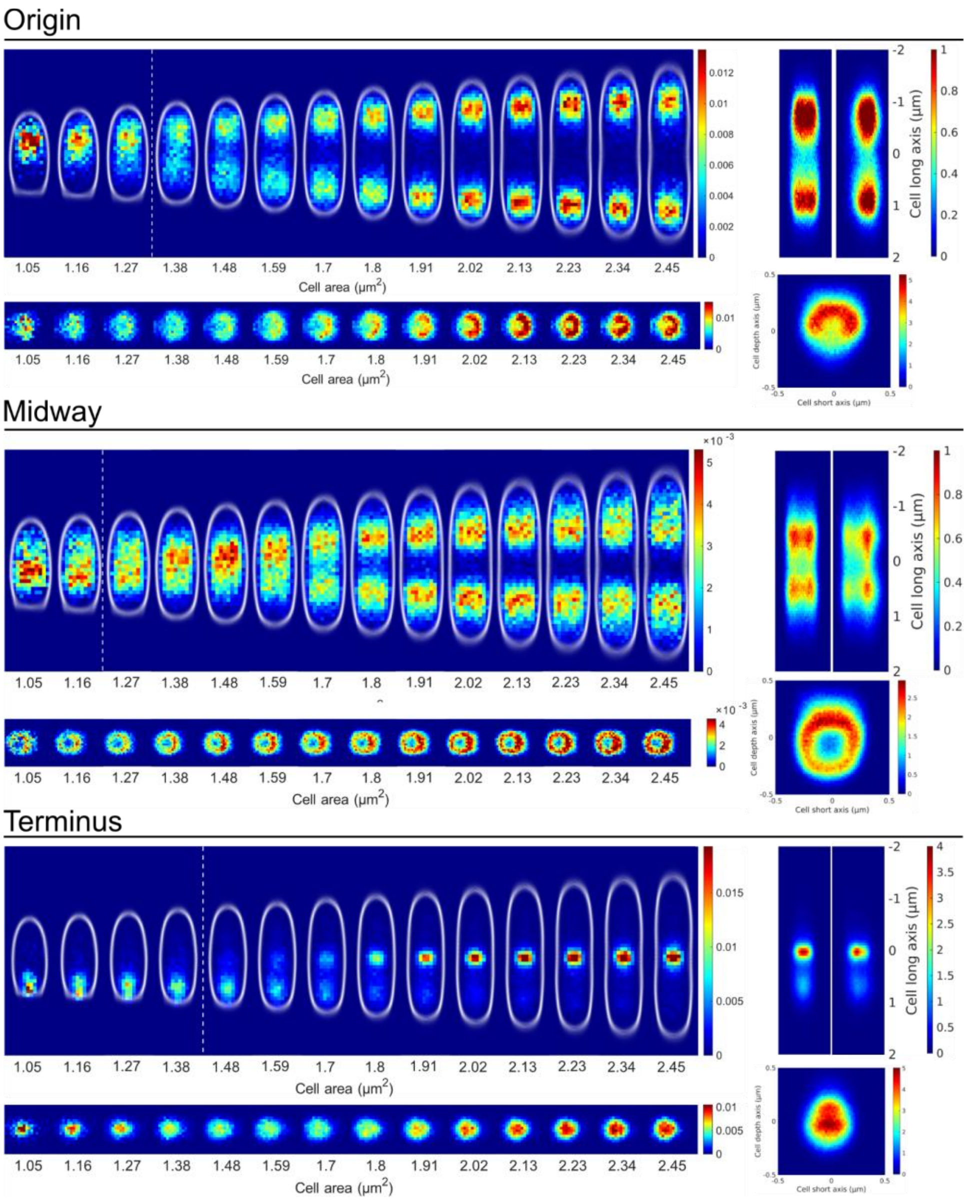

Supplementary Fig. 12: 3D location distributions of chromosomal loci. As in Fig. 3 but for repeated experiments with strains with fluorescently labeled loci.

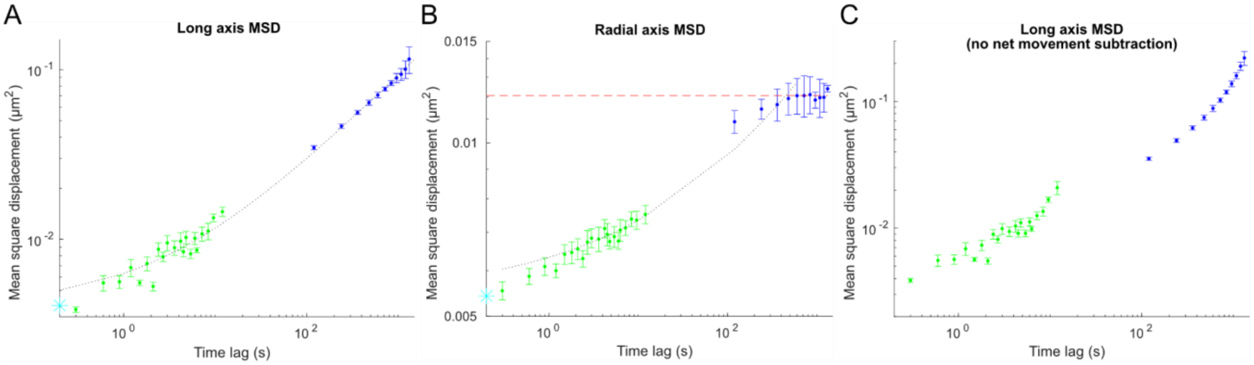

Supplementary Fig. 13. 3D MSD of the midway locus. MSD along **a,** long axis and **b,** radial axes as in Fig. 4a-b, but with fitting an exponential function to the MSD values (Materials and Methods). **c,** Long axis MSD as in Fig. 4a, but without subtraction of net movement due to cell growth prior to MSD estimation. Black dotted lines show an exponential function fit to the MSD values (Long axis slope (with 95% confidence interval): 0.5351 (0.5097, 0.5604). Radial axis slope (with 95% confidence interval): 0.3263 (0.2988, 0.3537)). Cyan stars show the y-axis intercept based on a linear fit to the first five MSD values as in Supplementary Fig. 15.

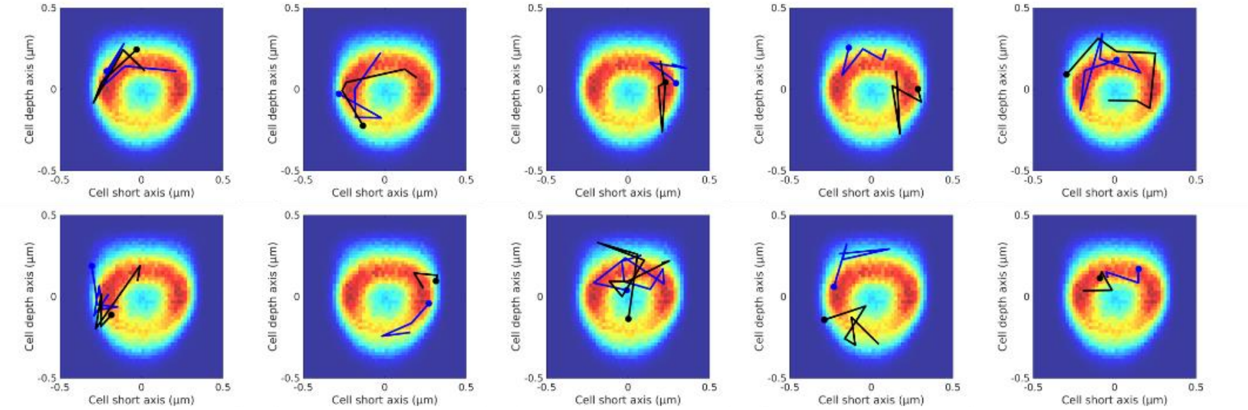

Supplementary Fig. 14: Examples of 3D chromosomal locus trajectories at the cell poles. As in Fig. 5b, but for two locus copies from other cells in the same experiment. The yz-plane distribution is the same as in Fig. 3b.

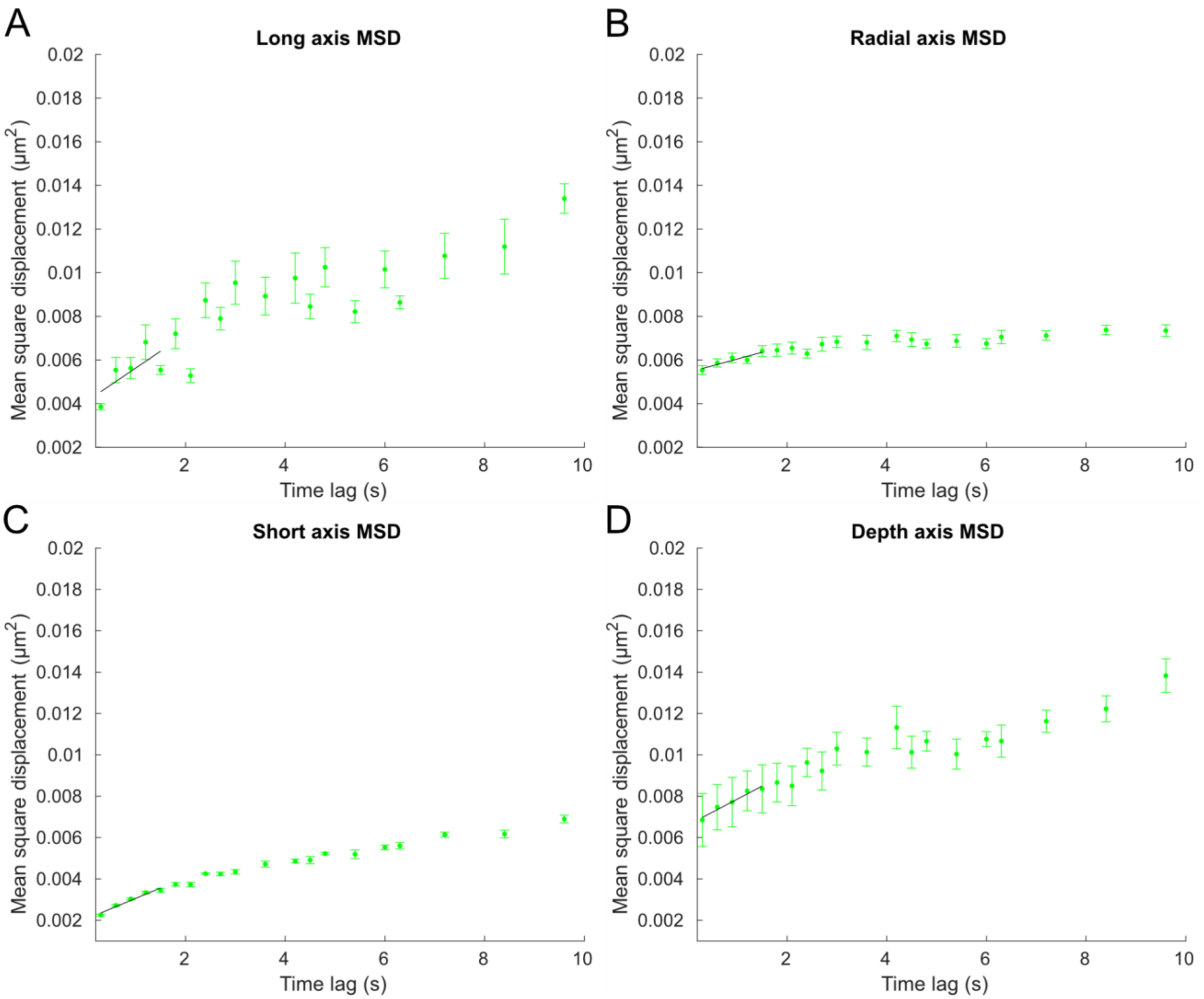

Supplementary Fig. 15: Localization error estimation from 3D locus tracking. Seconds time scale MSD along the cell's **a,** long, **b,** radial, **c,** short, and **d,** depth axes. Black lines show a line fit to the first five MSD values (Long axis intercept (with 95% confidence interval): 0.004058 (0.001186, 0.006931). Radial axis intercept: 0.005422 (0.00497, 0.005874). Short axis intercept (with 95% confidence interval): 0.002053 (0.001671, 0.002434). Depth axis intercept (with 95% confidence interval): 0.006591 (0.006088, 0.007094)).

A.11 Bacterial strains

Table S1. List of bacterial strains used in this study. For the chromosomal labels the number indicates the chromosomal base pair position where the label has been introduced.

Strain number	Genotype	Reference
EL3468	Eco MG1655 rph+ ΔmalI::frt<br>intC::P59-malI-SYFP2-frt<br>gtrA::P58-mCherry2-parB-SpR<br>3960236::KanR::MalOx12	This study
EL3513	Eco MG1655 rph+ ΔmalI::frt	This study

	intC::P59-malI-SYFP2-frt<br>gtrA::P58-mCherry2-parB-SpR<br>478731::KanR::MalOx12	
EL3464	Eco MG1655 rph+ ΔmalI::frt<br>intC::P59-malI-SYFP2-frt<br>gtrA::P58-mCherry2-parB-SpR<br>1637533::KanR::MalOx12	This study
EL2239	Eco BW25993 intC::P70-lacY-PAmCherry CmR	This study

Supplementary References

1. Kandavalli, V., Karempudi, P., Larsson, J. & Elf, J. Rapid antibiotic susceptibility testing and species identification for mixed samples. *Nat. Commun.* **13**, 1–8 (2022).
2. Fu, S. *et al.* Field-dependent deep learning enables high-throughput whole-cell 3D super-resolution imaging. *Nat. Methods* **20**, 459–468 (2023).
3. Liu, R. *et al.* An intriguing failing of convolutional neural networks and the CoordConv solution. *Adv. Neural Inf. Process. Syst.* **31**, (2018).
4. Speiser, A. *et al.* Deep learning enables fast and dense single-molecule localization with high accuracy. *Nat. Methods* **18**, 1082–1090 (2021).
5. Diekmann, R. *et al.* Photon-free (s)CMOS camera characterization for artifact reduction in high- and super-resolution microscopy. *Nat. Commun.* **13**, 1–9 (2022).
6. Vaughan Pratt Sun Microsystems, Inc. Direct least-squares fitting of algebraic surfaces. https://dl.acm.org/doi/10.1145/37401.37420 doi:10.1145/37401.37420.
7. Feed, F. C., Following, M. & Preferences, C. Circle Fit (Pratt method). https://se.mathworks.com/matlabcentral/fileexchange/22643-circle-fit-pratt-method.
