# Supplementary material for: Three-dimensional localization and tracking of chromosomal loci throughout the Escherichia coli cell cycle
Source: Commun Biol. 2024 Nov 5;7:1443. doi: 10.1038/s42003-024-07155-9 (PMC11538341; doi:10.1038/s42003-024-07155-9)
Supplement: Supplementary file 3 — Description of Additional Supplementary Files [file 42003_2024_7155_MOESM3_ESM.docx]

**Description of Additional Supplementary Files**

File name: Supplementary Data 1
Description: Statistics for the experiments performed in this study.

File name: Supplementary Data 2
Description: Statistics for two-dimensional histograms. The number of localized foci and detected cell for each cell area bin in the two-dimensional histograms.
